# Coagulation activation is associated with genomic-instability-related features in TP53-mutated AML and MDS: routine laboratory patterns beyond classical disseminated intravascular coagulation

**DOI:** 10.3389/fcell.2026.1918027

**Published:** 2026-08-21

**Authors:** Xiaoqin Xin, Chaoqiang Zheng, Jungao Huang, Qing Xie

**Affiliations:** 1 Department of Clinical Laboratory, Ganzhou People’s Hospital, Ganzhou, Jiangxi, China; 2 Department of Medical Genetics, Ganzhou Maternal and Child Health Hospital, Ganzhou, Jiangxi, China

**Keywords:** acute myeloid leukaemia, coagulation, d-dimer, disseminated intravascular coagulation, genomic instability, myelodysplastic syndromes, phenotypic clustering, TP53

## Abstract

**Background:**

Disseminated intravascular coagulation (DIC) is a serious complication of acute myeloid leukemia (AML) associated with poor prognosis. In TP53-mutated AML and myelodysplastic syndrome (MDS), however, the classical ISTH criteria rarely identify overt DIC, although bleeding and thrombotic complications are well documented in acute leukaemia. We hypothesized that these patients exhibit a lower-grade, subclinical coagulation activation that is associated with the underlying genomic-instability-related features of TP53-mutant disease.

**Methods:**

We retrospectively analyzed 107 consecutive patients with TP53-mutated AML (n = 52) or high-risk MDS (MDS, n = 55), median age 65 years, diagnosed and initially evaluated at our centre between 2018 and 2025. Seven routine coagulation markers and 46 co-mutated genes were evaluated for associations with overall survival (OS) using univariate and multivariable Cox regression, continuous dose–response modeling, and unsupervised k-means clustering. Internal validity was assessed by 1000 bootstrap resamples.

**Results:**

Overt DIC according to ISTH criteria was rare (15%). Subclinical activation was common: 50% of patients had a D-dimer ≥1 μg/mL, 41% a fibrinogen ≥4 g/L, and 29% an INR ≥1.2. In univariate analysis, D-dimer, fibrinogen, INR, prothrombin time, and activated partial thromboplastin time were each associated with OS (HR 1.33–1.38 per SD; all p < 0.05). Complex karyotype correlated with higher D-dimer (median 1.39 vs. 0.60 μg/mL, p = 0.022) and fibrinogen (3.91 vs. 2.53 g/L, p = 0.007), while TP53 variant allele frequency (VAF) showed modest positive correlations with D-dimer (ρ = 0.21), INR (ρ = 0.27), and PT (ρ = 0.27; all p < 0.05). Clustering identified three coagulation phenotypes: Silent (51%), Thrombo-inflammatory (31%), and Consumption-like (18%), showing a graded but statistically non-significant gradient in molecular features and a stepwise decline in median OS (14, 10 and 8 months; log-rank p = 0.041). After adjustment for complex karyotype, TP53 VAF, and favorable co-mutation count, the Consumption-like phenotype was associated with a non-significant increased risk (HR 1.83, 95% CI 0.92–3.65, p = 0.084), whereas favorable co-mutation pathways remained independently protective (HR 0.56, 95% CI 0.35–0.90, p = 0.016).

**Conclusion:**

In TP53-mutated AML/MDS, coagulation activation intensity is associated with the degree of genomic instability. The three phenotypes may add biological resolution beyond classical DIC and cytogenetic risk groups, but represent laboratory patterns rather than validated bleeding or thrombosis prediction tools. However, after accounting for genomic features, phenotypes were not independent predictors of outcome, with complex karyotype, TP53 VAF, and favorable co-mutation count driving prognosis. Because treatment intensity and other clinical confounders were not available, these survival associations are hypothesis-generating. Coagulation profiling remains inexpensive, widely accessible, and offers a practical window into disease biology that warrants prospective validation.

## Introduction

TP53 mutations are the most adverse genetic lesion in acute myeloid leukaemia (AML) and high-risk myelodysplastic syndromes (MDS): median overall survival is only 6–12 months, whether patients receive intensive chemotherapy, hypomethylating agents or, in most series, allogeneic transplantation (Grob et al., 2022; Bernard et al., 2020; Daver et al., 2022). Recent advances have refined the molecular classification of TP53-mutated disease. Integrating variant allele frequency (VAF) with 17p status enables discrimination between mono-allelic and multi-hit (bi-allelic) disease (Bernard et al., 2024; Tashakori et al., 2022). TP53 mutations are also strongly associated with complex karyotypes and aneuploidy, consistent with the loss of p53-mediated genomic surveillance (Rücker et al., 2012; Welch et al., 2016). By contrast, the clinical phenotype remains poorly characterised in the routine laboratory tests performed before treatment.

Coagulation is one aspect of this phenotype that has drawn remarkably little attention. Overt disseminated intravascular coagulation (DIC) is uncommon at AML diagnosis outside acute promyelocytic leukaemia, with classical ISTH criteria identifying it in only 10%–20% of patients (Taylor et al., 2001; Libourel et al., 2016). Lower-grade activation of the cascade is another matter: a rise in D-dimer or fibrin degradation products without frank fibrinogen consumption is increasingly viewed as a feature of aggressive leukaemia and a marker of inflammatory and endothelial activation (Falanga et al., 2013; Horowitz and Brenner, 2020; Rickles and Falanga, 2009).Whether this sub-clinical activation is mechanistically tied to TP53-driven genomic instability has, to our knowledge, never been examined systematically. Elevated D-dimer and fibrinogen are already known to carry adverse prognostic weight in AML, but these associations have generally been reported without reference to the underlying molecular lesion and in morphologically or cytogenetically defined cohorts rather than in a uniformly TP53-mutated population. What has not been established is whether the intensity of routine coagulation activation is associated with the specific genomic-instability programme of TP53-mutated disease, and whether unsupervised coagulation phenotypes derived from inexpensive routine tests carry biological or prognostic meaning within this uniquely high-risk and biologically distinct subset.

Several observations make such a link plausible. TP53 loss disrupts endothelial homeostasis and drives procoagulant tissue factor expression in malignant cells (Kumar et al., 2011; Yokoyama et al., 2019),and aneuploid blasts shed more procoagulant microvesicles and inflammatory cytokines than diploid clones (Geddings and Mackman, 2013). Fibrinogen, an acute-phase reactant, is an established independent prognostic marker across several AML series (Ding et al., 2023), yet what it reflects biologically within molecular subgroups is unknown.

We therefore hypothesised that, in TP53-mutated AML and MDS, coagulation activation at diagnosis is predominantly subclinical rather than overt, and that its intensity is associated with the underlying degree of TP53-driven genomic instability. To examine this, we assembled a single-centre cohort of 107 consecutive patients with TP53-mutated AML or MDS, each with paired molecular profiling and a pre-treatment coagulation panel. We characterised the prevalence of overt DIC and of lower-grade coagulation activation, related routine coagulation markers to cytogenetic and molecular indices of genomic instability, applied unsupervised clustering to define coagulation phenotypes, and evaluated whether these markers and phenotypes provided prognostic information independent of established cytogenetic and molecular risk factors.

## Methods

### Patients and inclusion criteria

We retrospectively identified all consecutive patients with newly diagnosed AML or MDS who carried at least one TP53 mutation on next-generation sequencing (NGS) and were treated at our centre between 2018 and 2025. All remaining cases were independently reviewed by two hematopathologists according to the WHO/ICC classification criteria. AML was defined by bone marrow blast percentage ≥20%. For MDS, inclusion criteria were: (a) MDS with excess blasts (MDS-EB, 5%–19% blasts); and (b) MDS with <5% blasts but harboring high-risk cytogenetic abnormalities (e.g., complex karyotype, −7/del(7q)) and/or TP53 mutations, given their similarly adverse prognosis to MDS-EB. We excluded patients with acute promyelocytic leukaemia (PML–RARA fusion) and those with an active competing second malignancy. The study was approved by the institutional review board (approval number: PJB2025-145-01); because it relied on retrospective record review, the requirement for informed consent was waived, in keeping with the Declaration of Helsinki. AML and MDS were analysed as a single cohort because, in the TP53-mutated and predominantly multi-hit state, the two diagnoses share the same p53-deficient, genomically unstable biology and behave as a clinical and molecular continuum. To limit disease-type confounding, AML-versus-MDS status examined as a subgroup analysis (Supplementary Table S1).

### Clinical, molecular and coagulation variables

From the electronic records, we pulled pre-treatment variables including demographics, complete blood count, bone marrow blast percentage, diagnostic category (AML versus MDS), karyotype (with complex karyotype defined as three or more unrelated cytogenetic abnormalities), and the routine coagulation panel. That panel covered prothrombin time (PT), international normalised ratio (INR), activated partial thromboplastin time (APTT), thrombin time (TT), fibrinogen, D-dimer, and platelet count. Coagulation tests were performed on citrate plasma by standard automated coagulometry. D-dimer was measured by an immunoturbidimetric assay and reported in fibrinogen-equivalent units (FEU; µg/mL). A single analytic platform with reagent kits from one manufacturer and unchanged manufacturer reference ranges was used throughout the study period, and routine internal and external quality-control programmes confirmed analytic stability across 2018–2025. Molecular testing was performed on bone marrow aspirate using a targeted NGS panel covering 46 myeloid relevant genes. We recorded the TP53 VAF of the dominant mutation. For multi-hit TP53 status, we approximated it with two surrogate definitions applied in parallel. V2 was defined as complex karyotype or TP53 VAF >50%. The strict definition required both complex karyotype and TP53 VAF >50%. We used V2 as the primary stratifier. Because centralised 17p-deletion and allele-specific testing were not uniformly available, this surrogate relies on karyotype and VAF rather than on direct assessment of bi-allelic status and may therefore misclassify a minority of patients. Co-mutated genes were grouped into five functional pathways, following earlier classifications (Papaemmanuil et al., 2016; Tazi et al., 2022): DNA methylation (*TET2, DNMT3A, IDH1, IDH2*); spliceosome (*SF3B1, U2AF1, SRSF2, SF3A1*); RAS/signalling (*FLT3-ITD, FLT3-TKD, KRAS, NRAS, PTPN11, NF1, JAK2, CSF3R*); transcription factors (*RUNX1, NPM1, CEBPA, WT1, GATA2, ETV6*); and chromatin remodellers (*ASXL1, ASXL2, BCOR, PHF6, EZH2, KMT2A, KMT2C, KMT2D, SETD2*; nine genes, each patient counted once even when carrying several mutations in the pathway). The primary endpoint was overall survival (OS), measured from diagnosis to death from any cause, with patients alive at last contact censored. Progression-free survival (PFS) was measured from diagnosis to documented progression or relapse, whichever came first. Median follow-up was estimated by the reverse Kaplan-Meier method.

### Statistical analysis: descriptive and univariate

Continuous variables were summarised as median (interquartile range, IQR) and compared with the Mann–Whitney U or Kruskal–Wallis test; categorical variables were summarised as n (%) and compared with the chi-square or Fisher exact test. Associations between continuous variables and ordinal molecular features were assessed by Spearman correlation. Survival was estimated by the Kaplan-Meier method and compared by the log-rank test, with hazard ratios and 95% confidence intervals (CIs) from Cox proportional-hazards regression. For univariate Cox models, coagulation markers were standardised so that hazard ratios are expressed per 1 standard deviation; skewed markers (D-dimer, APTT, platelet count) were log-transformed before standardisation. The dose–response between D-dimer and hazard was shown using quintile reference categories with a log-linear Cox overlay, and tested for non-linearity with a cubic Cox model. The proportional-hazards assumption was checked by comparing hazard ratios in time strata split at median follow-up. D-dimer was available in 100 of the 107 patients; the seven missing values arose because the assay had not been ordered at diagnosis in a small number of retrospectively identified cases, a pattern that was unrelated to disease type, cytogenetic risk or survival and therefore consistent with data missing at random. All analyses involving D-dimer, the coagulation clustering and the TPC-S were accordingly performed on complete cases (n = 100, 68 deaths), and effect estimates were consistent between the full and complete-case samples and across the pre-specified sensitivity analyses (Supplementary Tables S1, S2).

### Unsupervised phenotypic clustering and the TPC-S score

For clustering, six coagulation markers (D-dimer, fibrinogen, INR, PT, APTT and platelet count, log-transformed as above) were standardised to zero mean and unit variance. We ran k-means clustering with 100 random initialisations and evaluated k = 2, 3 and 4. The number of clusters was chosen by combining the silhouette coefficient with clinical interpretability, settling on k = 3 (silhouette 0.22 for k = 3, vs. 0.26 for k = 2 and 0.18 for k = 4). Clusters were relabelled by ascending median D-dimer (Cluster A = Silent, Cluster B = Thrombo-inflammatory, Cluster C = Consumption-like). Principal component analysis (PCA) was used for visualisation only. We also pre-specified a composite Thrombo-Phenotype Coagulation Score (TPC-S, range 0–5) from five binary thresholds taken from earlier literature: D-dimer ≥2 μg/mL (+1), D-dimer ≥4 μg/mL (+1), fibrinogen ≥4.5 g/L (+1), INR ≥1.2 (+1) and PT ≥ 14 s (+1). Each threshold was pre-specified from prior coagulation and DIC literature rather than derived from the present data: the two D-dimer cut-offs (≥2 and ≥4 μg/mL) mark moderate and marked fibrin turnover, fibrinogen ≥4.5 g/L captures acute-phase elevation, and INR ≥1.2 and PT ≥ 14 s denote early extrinsic-pathway prolongation. Because prothrombin time and INR were almost perfectly collinear (ρ = 0.99) and the two D-dimer thresholds load on a single fibrin-turnover axis, we also pre-specified a parsimonious three-component variant (simplified TPC-S, range 0–3: D-dimer ≥2 μg/mL, fibrinogen ≥4.5 g/L, and INR ≥1.2) that avoids double-counting D-dimer and retains only INR. Sensitivity analyses examined this simplified score and repeated the clustering after excluding PT and retaining INR alone (Supplementary Table S2).

### Multivariable modelling and internal validation

Multivariable Cox models were fitted on the same complete-case subset (n = 100, 68 events. Those patients with complete coagulation panels, who could therefore be assigned to a cluster, so that the four models could be compared directly. Model A included only clinical variables (age ≥65 years, AML vs. MDS, complex karyotype and TP53 VAF per 10%). Model B added the favourable co-mutation pathway count to this baseline; Model C instead added the TPC-S score; and Model D combined the baseline with both the favourable pathway count and the coagulation-cluster indicators. Discrimination was summarised by Harrell’s C-index, and the models were internally validated by a 1000-resample bootstrap with optimism correction. We applied no formal correction for multiple testing to the molecular subgroup analyses, which were exploratory; these associations should be read as hypothesis-generating. Given the large number of exploratory comparisons performed across clinical and molecular subgroups, co mutation pathways, and marker correlations, we did not adjust for multiplicity. Consequently, individual nominal p values, especially those approaching the 0.05 threshold, carry a considerable risk of false positive findings. These results should therefore be considered exploratory unless they are validated in independent datasets. Treatment-related variables (induction intensity; exposure to hypomethylating agents, venetoclax, intensive chemotherapy or allogeneic transplantation; and supportive-care-only management) and other major clinical confounders (performance status, infection at diagnosis, and renal or hepatic dysfunction) were not uniformly recorded and could not be modelled. The survival analyses are therefore adjusted only for the pre-specified cytogenetic and molecular covariates and should be regarded as hypothesis-generating rather than as validated independent prognostic models. All analyses were performed using Python 3.12. Cox proportional-hazards partial-likelihood estimation and Kaplan-Meier survival estimators were implemented for survival analyses. Unsupervised clustering was conducted with scikit-learn version 1.4, and non-parametric statistical tests were performed using SciPy version 1.13. The study is reported in line with the STROBE guidelines for observational studies.

## Results

### Cohort characteristics

One hundred and seven patients met the inclusion criteria (Table 1). AML (n = 52, 48.6%) and MDS (n = 55, 51.4%) were represented about equally; the median age was 65 years (IQR 58–71), and 54% were male. A complex karyotype was present in 78 patients (73%), median TP53 VAF was 51% (IQR 28%–77%), and 78% met the V2 multi-hit definition (complex karyotype OR VAF >50%). Co-mutations in at least one of the five functional pathways were found in 64% of patients (68/107), most often in DNA-methylation genes (31%), RAS/signalling (23%) and chromatin remodellers (19.6%; 21 patients). At a median follow-up of 22 months (reverse Kaplan–Meier), 72 patients (67%) had died, and median overall survival for the whole cohort was 12 months.

**TABLE 1:** Baseline clinical, molecular and coagulation characteristics, overall and by coagulation phenotype cluster.

Variable	Overall (n = 107)	Silent (n = 51)	Thrombo-inflammatory (n = 31)	Consumption-like (n = 18)	P (Kruskal/χ^2^)
Total (n)	107	51	31	18	​
Age, median (IQR)	65 (58–71)	64 (55–71)	65 (59–70)	64 (54–70)	1.000
Age ≥65, n (%)	55 (51.4)	25 (49.0)	17 (58.1)	9 (50.0)	0.874
Male sex, n (%)	58 (54.2)	20 (39.2)	20 (64.5)	14 (77.8)	0.007
AML (vs. MDS), n (%)	52 (48.6)	23 (45.1)	18 (58.1)	8 (44.4)	0.477
Blast %, median (IQR)	15 (6–46)	13 (4–50)	22 (12–46)	14 (6–40)	0.393
Molecular/cytogenetic					
Complex karyotype, n (%)	78 (72.9)	32 (62.7)	25 (80.6)	15 (83.3)	0.107
TP53 VAF (%), median (IQR)	50.84 (28.10–77.05)	48.00 (20.70–71.80)	45.00 (31.84–73.36)	69.53 (44.75–86.05)	0.096
Multi-hit proxy (V2), n (%)	83 (77.6)	35 (68.6)	25 (80.6)	17 (94.4)	0.069
Multi-hit (strict), n (%)	49 (45.8)	21 (41.2)	14 (45.2)	11 (61.1)	0.343
DNA methylation co-mut, n (%)	33 (30.8)	13 (25.5)	10 (32.3)	7 (38.9)	0.536
Spliceosome co-mut, n (%)	10 (9.3)	6 (11.8)	3 (9.7)	0 (0.0)	​
RAS/signaling co-mut, n (%)	25 (23.4)	13 (25.5)	8 (25.8)	4 (22.2)	​
Transcription co-mut, n (%)	16 (15.0)	8 (15.7)	6 (19.4)	2 (11.1)	​
Chromatin co-mut, n (%)	21 (19.6)	9 (17.6)	8 (25.8)	3 (16.7)	​
≥1 favorable pathway, n (%)	38 (35.5)	18 (35.3)	13 (41.9)	5 (27.8)	0.83
Coagulation (median, IQR)					
D-dimer (µg/mL)	1.02 (0.46–2.72)	0.62 (0.29–0.93)	2.16 (1.08–3.42)	2.72 (1.60–8.38)	<0.001
Fibrinogen (g/L)	3.64 (2.42–4.86)	2.53 (2.25–3.30)	5.04 (4.60–5.75)	3.77 (2.06–4.46)	<0.001
INR	1.12 (1.02–1.21)	1.03 (0.97–1.10)	1.15 (1.12–1.21)	1.27 (1.25–1.35)	<0.001
PT (s)	12.90 (11.80–13.80)	12.00 (11.30–12.80)	13.30 (12.85–13.85)	14.50 (14.20–15.45)	<0.001
APTT (s)	26.60 (24.60–29.15)	24.70 (22.80–26.70)	28.10 (26.30–28.75)	34.20 (30.80–36.00)	<0.001
PLT (×10^9^/L)	37.00 (23.00–71.50)	48.00 (28.50–77.00)	38.00 (24.00–108.00)	28.50 (15.50–38.00)	0.062
Outcome					
OS event, n (%)	72 (67.3)	32 (62.7)	23 (74.2)	13 (72.2)	0.511
Median OS, months	12	14	10	8	0.051

IQR, interquartile range. P-values from Kruskal–Wallis (continuous) or chi-square (categorical) tests comparing the three clusters; categorical p reported only where all cells ≥5. Multi-hit (V2) defined as complex karyotype OR TP53 VAF >50%; multi-hit (strict) defined as both. Favorable pathway = ≥1 mutation in spliceosome, chromatin or transcription factor genes.

### Coagulation phenotype landscape—beyond classical DIC criteria

**FIGURE 1:**
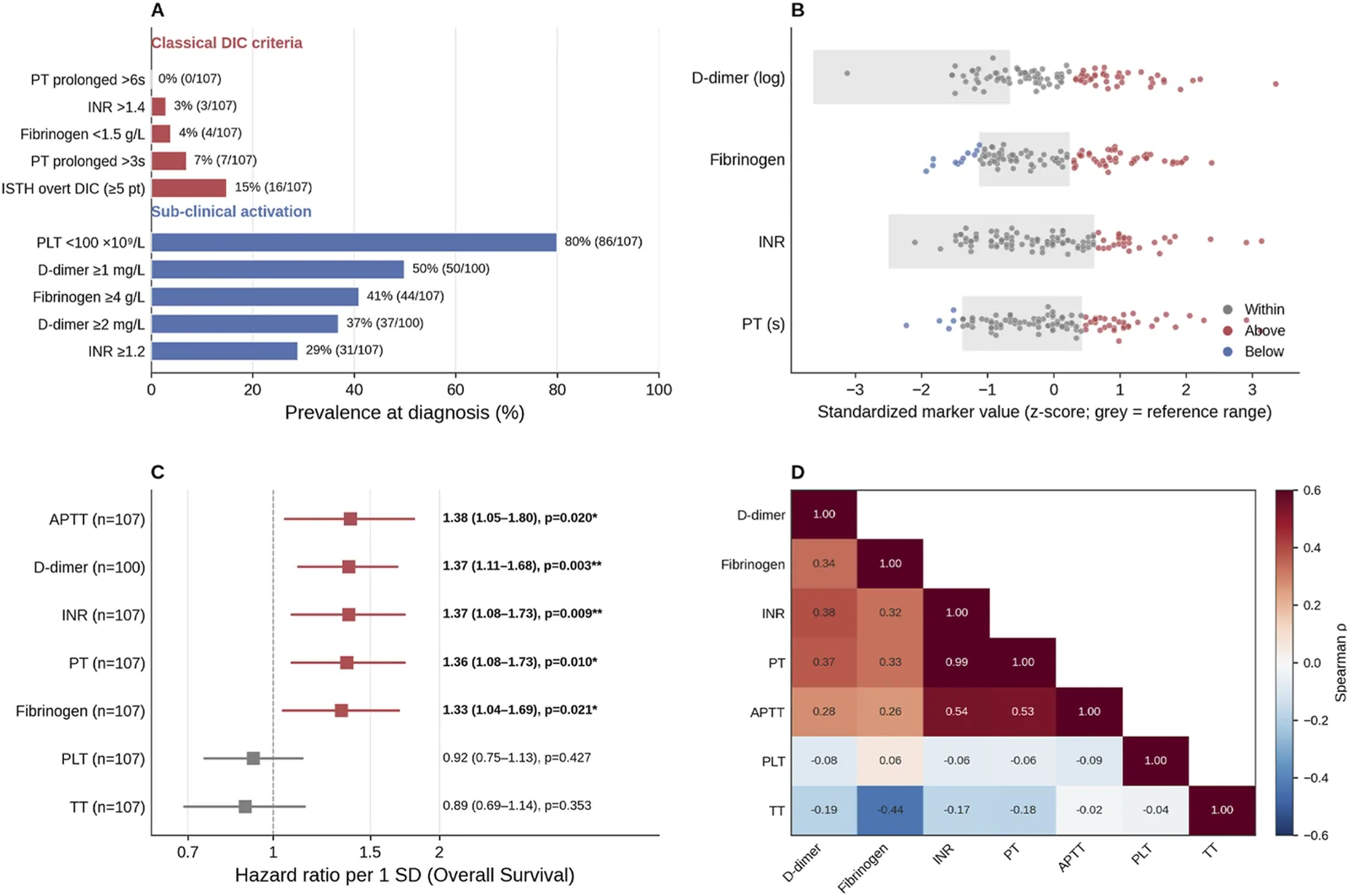
Baseline coagulation profile in TP53-mutated AML/MDS. **(A)** Prevalence of classical DIC abnormalities (red) and subclinical coagulation activation (blue), presented as percentage (n/N). Overt DIC was defined as an ISTH score ≥5. **(B)** Standardised distributions of D-dimer, fibrinogen, INR, and PT; colours indicate values above, within, or below the reference interval. **(C)** Univariable Cox associations with OS, expressed as HRs per 1-SD increase with 95% CIs. D-dimer, APTT, and platelet count were log-transformed. **(D)** Pairwise Spearman correlations. D-dimer was available for 100 patients and other markers for 107.

Only 16 patients (15%) fulfilled the ISTH criteria for overt DIC (score ≥5). Abnormalities in individual coagulation parameters were also uncommon, with no cases of PT prolongation ≥6 s, prolongation >3 s in only 7% (7/107), fibrinogen <1.5 g/L in 4% (4/107), and INR >1.4 in 3% (3/107) of patients (Figure 1A). Sub-clinical activation, by contrast, was common at diagnosis: D-dimer (available in 100 of 107 patients) was ≥1 μg/mL in 50% (50/100) and ≥2 μg/mL in 37% (37/100), fibrinogen was raised (≥4 g/L) in 41%, and INR was ≥1.2 in 29%. Thrombocytopenia (platelets <100 ×10^9^/L) was present in 80%. All four distributions were shifted towards higher values, but only D-dimer lay above its reference range in the majority of patients (72%); for fibrinogen, INR and PT most individual values remained within or below the reference interval (Figure 1B). On univariate Cox regression, five of the seven markers were associated with OS (p < 0.05): APTT (log, HR 1.38, 95% CI 1.05–1.80, p = 0.020), D-dimer (log, HR 1.37, 1.11–1.68, p = 0.003), INR (HR 1.37, 1.08–1.73, p = 0.009), PT (HR 1.36, 1.08–1.73, p = 0.010) and fibrinogen (HR 1.33, 1.04–1.69, p = 0.021); platelet count and thrombin time were not (Figure 1C). PT and INR were almost perfectly collinear (ρ = 0.99) but only modestly correlated with D-dimer (ρ = 0.37–0.38; Figure 1D).

### Coagulation activation is associated with markers of TP53 genomic instability

**FIGURE 2:**
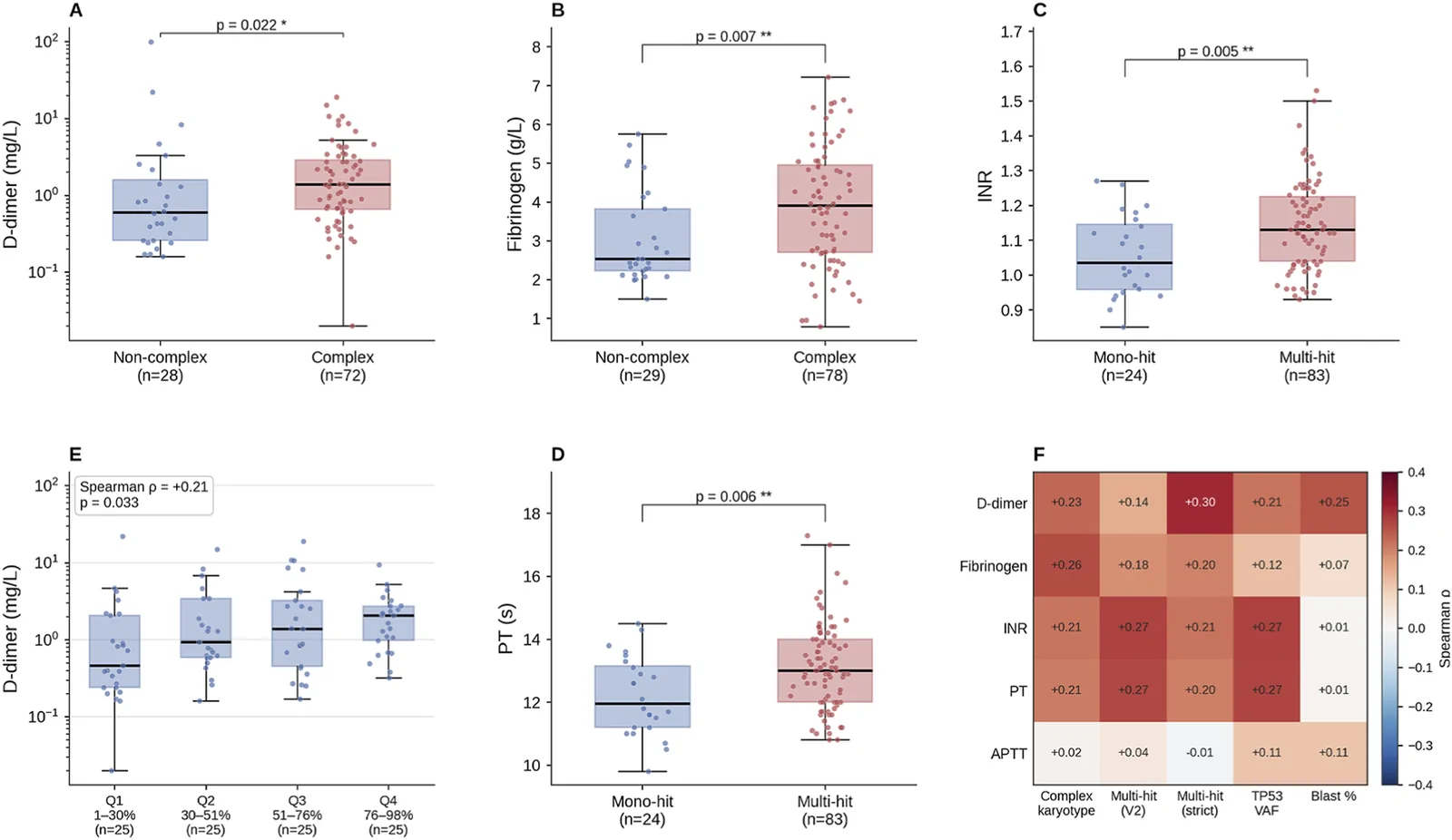
Associations between coagulation measures and genomic-instability-related features. **(A,B)** D-dimer and fibrinogen by complex-karyotype status. **(C,D)** INR and PT by TP53 multi-hit proxy (V2) status. **(E)** D-dimer across TP53 VAF quartiles; ρ and P were calculated using continuous values. **(F)** Spearman correlations between coagulation measures and cytogenetic, molecular, and blast-related features. Boxes show medians and interquartile ranges. V2 was defined as complex karyotype or TP53 VAF >50%; the strict proxy required both.

Patients with a complex karyotype had higher D-dimer (median 1.39 vs. 0.60 μg/mL, n = 72 vs. 28, p = 0.022; Figure 2A) and fibrinogen (3.91 vs. 2.53 g/L, p = 0.007; Figure 2B). V2 multi-hit patients had higher INR (1.13 vs. 1.04, p = 0.005; Figure 2C) and longer PT (13.0 vs. 11.95 s, p = 0.006; Figure 2D). TP53 VAF rose with D-dimer across quartiles (Spearman ρ = +0.21, p = 0.033; Figure 2E), the top VAF quartile showing an approximately 4.5-fold higher median D-dimer than the bottom (2.07 vs. 0.46 μg/mL). In the cross-association heatmap (Figure 2F), coagulation parameters showed the strongest associations with genomic instability features, including strict multi-hit status, complex karyotype, and TP53 VAF. Among these markers, D-dimer showed the single strongest association, with strict multi-hit status (ρ = +0.30), and correlations ranging from +0.14 to +0.30 across the genomic-instability indices; fibrinogen was most closely related to complex karyotype (ρ = +0.26), whereas INR and PT correlated most strongly with multi-hit (V2) status and TP53 VAF (both ρ = +0.27). APTT showed only weak correlations with molecular characteristics (all |ρ| ≤ 0.11).

### Three coagulation phenotypes emerge from unsupervised clustering

**FIGURE 3:**
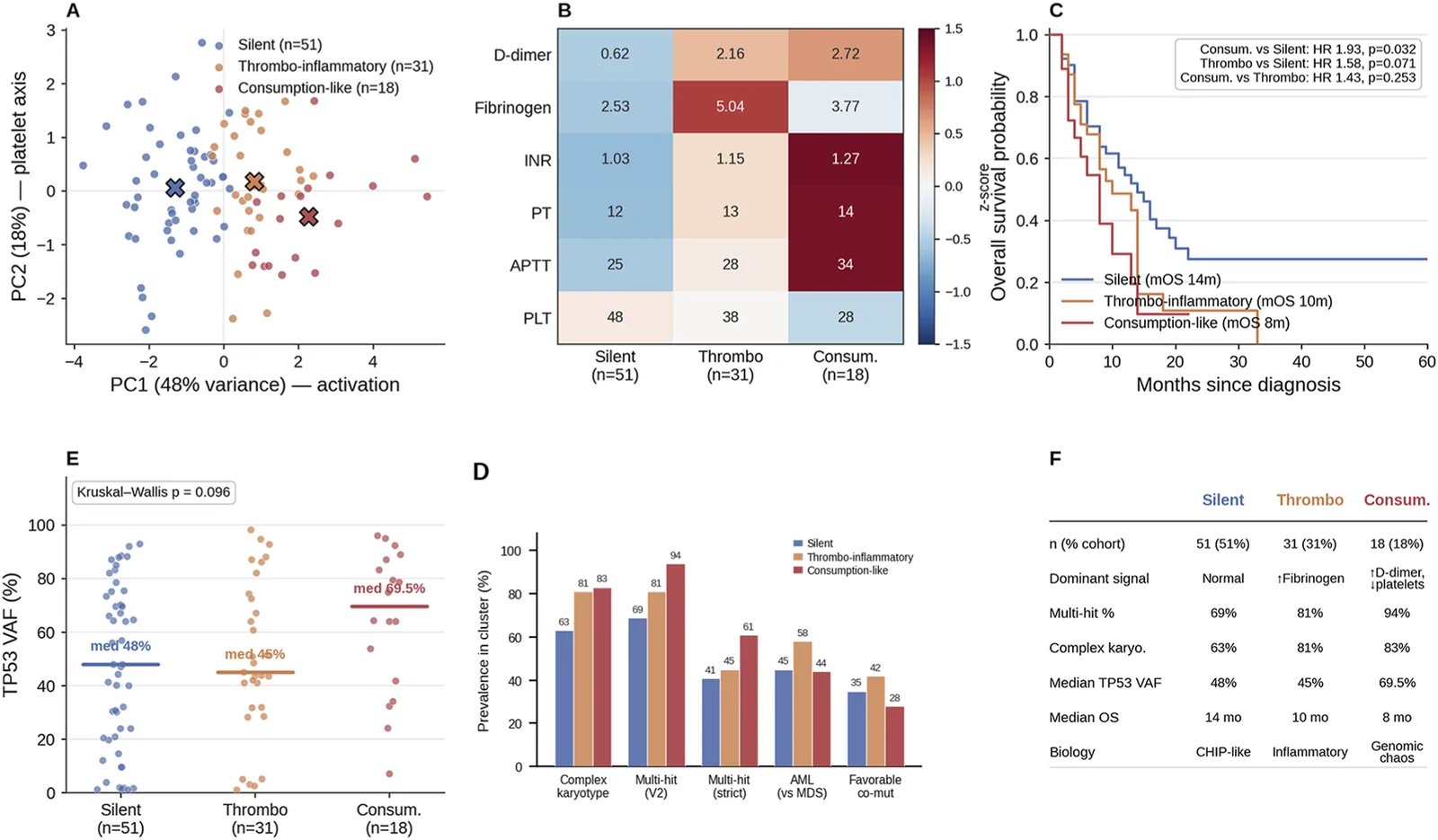
Unsupervised clustering identifies three exploratory coagulation phenotypes. K-means clustering of six standardised markers in 100 complete cases identified Silent (n = 51), Thrombo-inflammatory (n = 31), and Consumption-like (n = 18) phenotypes. **(A)** PCA projection; crosses indicate cluster centres. **(B)** Cluster median profiles. **(C)** Kaplan–Meier OS curves with pairwise Cox HRs and P values. **(D)** Molecular and diagnostic composition. **(E)** TP53 VAF by cluster. **(F)** Descriptive phenotype summary. These clusters represent exploratory laboratory patterns rather than validated clinical classifications.

Among the 100 patients with complete coagulation profiling, k-means clustering identified three subgroups (Figure 3A; silhouette 0.22 for k = 3). The Silent phenotype (Cluster A, n = 51, 51%) had a median D-dimer of 0.62 μg/mL, near-normal fibrinogen (2.53 g/L), INR (1.03) and PT (12.0 s), and the highest platelet count (median 48 ×10^9^/L; Figure 3B). The Thrombo-inflammatory phenotype (Cluster B, n = 31, 31%) had moderately raised D-dimer (2.16 μg/mL) but markedly raised fibrinogen (5.04 g/L), with otherwise modest abnormalities. The Consumption-like phenotype (Cluster C, n = 18, 18%) had the highest D-dimer (2.72 μg/mL), globally prolonged coagulation times (INR 1.27, PT 14.5 s, APTT 34.2 s) and the lowest platelet count (28 ×10^9^/L). This cluster was designated Consumption-like because the combination of the highest D-dimer, globally prolonged clotting times and the lowest platelet count reproduces the laboratory signature of a low-grade consumptive coagulopathy, without necessarily implying overt disseminated intravascular coagulation.

Median OS was 14 months in Silent, 10 in Thrombo-inflammatory and 8 in Consumption-like (pairwise log-rank: Consumption-like vs. Silent HR 1.93, 95% CI 1.06–3.50, p = 0.032; Thrombo-inflammatory vs. Silent HR 1.58, 0.96–2.59, p = 0.071; Figure 3C). Cluster molecular composition followed the same gradient, although none of the differences reached statistical significance (Figure 3D): the proportion of multi-hit patients rose from 69% (Silent) to 81% (Thrombo-inflammatory) to 94% (Consumption-like), and complex karyotype from 63% to 81%–83% (p = 0.069 and p = 0.107, respectively; Table 1). Median TP53 VAF was higher in Consumption-like (69.5%) than in Silent (48%) or Thrombo-inflammatory (45%) clusters (Kruskal–Wallis p = 0.096; Figure 3E). The defining coagulation, molecular and survival characteristics of the three phenotypes are summarized in Figure 3F. The distribution of AML and MDS did not differ across clusters (MDS 54.9%, 41.9% and 55.6% in the Silent, Thrombo-inflammatory and Consumption-like clusters, respectively; p = 0.477).

### Co-mutation pathway context and outcome

**FIGURE 4:**
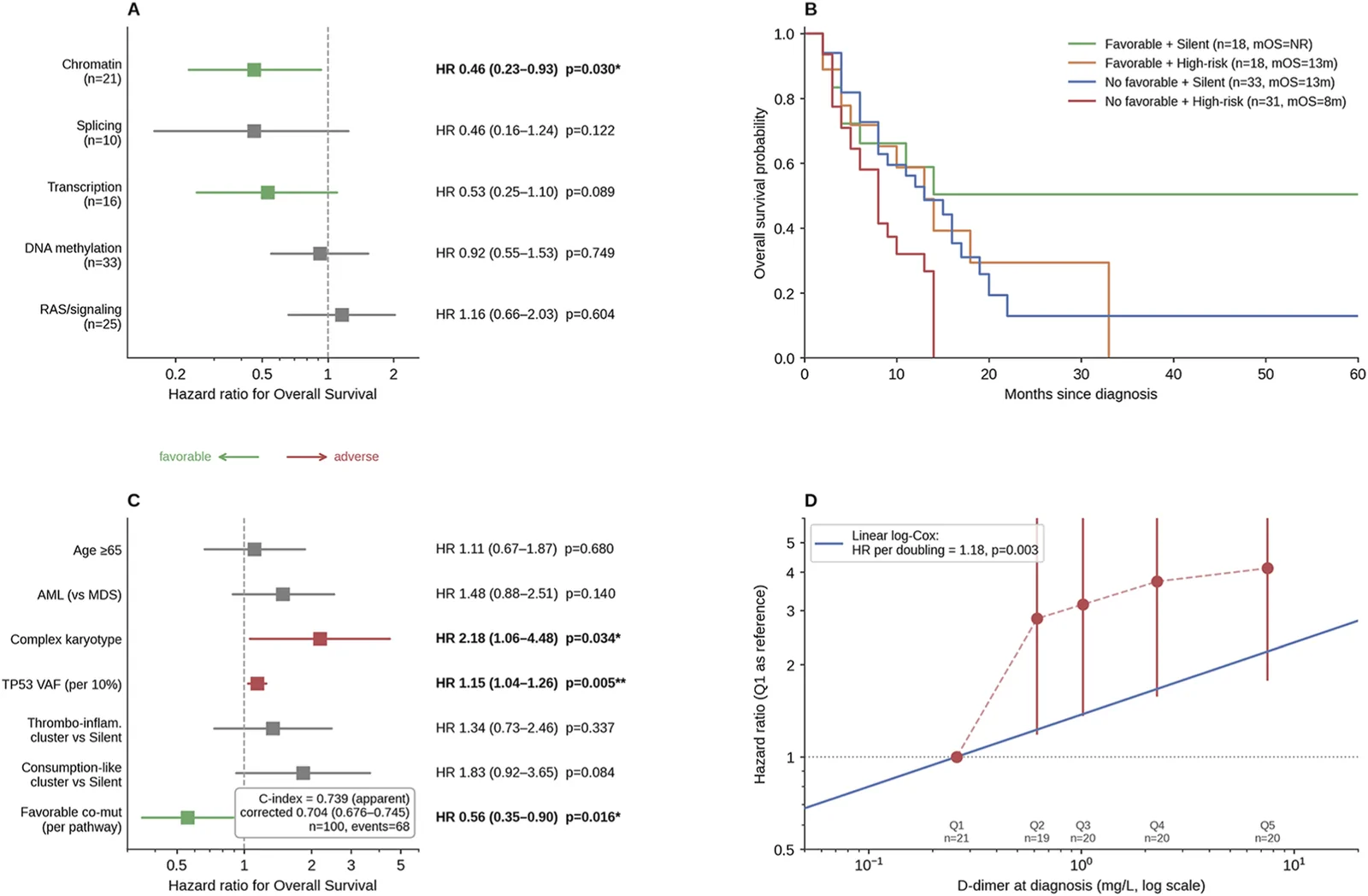
Co-mutation context and integrated prognostic analyses. **(A)** Univariable Cox associations between co-mutation pathways and OS. **(B)** Kaplan–Meier curves combining favourable-pathway status with Silent versus high-risk phenotype. **(C)** Multivariable Cox model in 100 patients with 68 deaths; the Silent phenotype was the reference. Apparent and bootstrap-corrected C-indices are shown. **(D)** D-dimer dose–response, showing quintile-specific HRs relative to Q1 and the log-linear Cox fit.

By individual pathway (Figure 4A), chromatin co-mutations (21 patients, 19.6%) were associated with better OS (HR 0.46, 95% CI 0.23–0.93, p = 0.030); similar trends were seen for spliceosome (n = 10, HR 0.46, 0.16–1.24, p = 0.122) and transcription-factor (n = 16, HR 0.53, 0.25–1.10, p = 0.089) co-mutations. We grouped these as the “favourable co-mutation pathways”; their cumulative count was protective on univariable analysis (HR per pathway 0.52, 95% CI 0.34–0.80, p = 0.003). DNA-methylation (n = 33, HR 0.92, 95% CI 0.55–1.53, p = 0.749) and RAS/signalling (n = 25, HR 1.16, 0.66–2.03, p = 0.604) co-mutations were prognostically neutral. Favourable pathway count did not significantly affect the distribution of D-dimer (Kruskal–Wallis p = 0.157) or fibrinogen (p = 0.188) (Supplementary Figure S1) or cluster assignment. Survival curves combining favourable-pathway status with the binary cluster (Silent vs. high-risk) ran from best (favourable-pathway/Silent) to worst (no-favourable-pathway/high-risk-cluster; Figure 4B).

### Univariate prognostic value of coagulation markers

In the total cohort of 107 patients with TP53-mutated AML/MDS (n = 100 for D-dimer), univariate Cox regression for overall survival (OS) showed that each one-standard-deviation (SD) increase in log-D-dimer, fibrinogen, INR, PT, and log-APTT was significantly associated with worse OS, with hazard ratios (HRs) ranging from 1.33 to 1.38 (all p < 0.05; Table 2). For progression-free survival (PFS), significant associations were maintained for D-dimer (HR 1.29, p = 0.0086), INR (HR 1.41, p = 0.0023) and PT (HR 1.41, p = 0.0030), but not for fibrinogen or APTT (Table 2). Platelet count and thrombin time (TT) showed no prognostic signal for either endpoint.

**TABLE 2:** Univariate Cox proportional hazards regression for coagulation markers vs. overall and progression-free survival.

Marker	n	HR for OS (95% CI)	P (OS)	HR for PFS (95% CI)	P (PFS)
D-dimer (log, per SD)	100	1.37 (1.11–1.68)	0.0033	1.29 (1.07–1.56)	0.0086
Fibrinogen (per SD)	107	1.33 (1.04–1.69)	0.0207	1.19 (0.93–1.51)	0.1613
INR (per SD)	107	1.37 (1.08–1.73)	0.0091	1.41 (1.13–1.77)	0.0023
PT (per SD)	107	1.36 (1.08–1.73)	0.0103	1.41 (1.12–1.76)	0.0030
APTT (log, per SD)	107	1.38 (1.05–1.80)	0.0201	1.22 (0.92–1.62)	0.1757
PLT (log, per SD)	107	0.92 (0.75–1.13)	0.4275	0.94 (0.77–1.14)	0.5033
TT (per SD)	107	0.89 (0.69–1.14)	0.3526	0.89 (0.72–1.12)	0.3293

HR, expressed per 1 standard deviation increase of the marker (log-transformed where indicated). PFS, progression-free survival.

### Multivariable integration and continuous dose-response

We fitted four Cox models on the same 100 patients with complete coagulation profiling (68 events; Table 3). In the clinical baseline (Model A), only complex karyotype (HR 2.41, 95% CI 1.17–4.97, p = 0.017) and TP53 VAF per 10% (HR 1.15, 1.05–1.26, p = 0.003) were independent predictors (apparent C-index 0.734; optimism-corrected 0.717, 95% CI 0.682–0.740). Adding favourable pathway count (Model B) gave an independent protective effect (HR 0.57, 0.36–0.91, p = 0.017) without weakening the cytogenetic or molecular signals. Adding TPC-S instead (Model C) gave a significant independent association (HR 1.24 per +1 point, 1.05–1.47, p = 0.013), although the optimism-corrected C-index was essentially unchanged (0.718 vs. 0.717 for Model A). In pre-specified sensitivity analyses, the parsimonious three-component score (simplified TPC-S) showed a consistent direction and magnitude of effect (HR 1.30 per +1 point, 95% CI 1.01–1.67, p = 0.038), and repeating the clustering and score using INR alone (excluding the collinear PT) reassigned only 4 of 100 patients (4.0%; adjusted Rand index 0.879, Cohen’s κ 0.935) and did not materially change the pattern of survival associations (high-risk versus silent phenotype HR 1.80, 95% CI 1.10–2.94, versus 1.82, 95% CI 1.11–2.98 in the primary analysis; Supplementary Table S2).

**TABLE 3:** Multivariable Cox proportional hazards models for overall survival.

Variable	Model A	Model B	Model C	Model D
	Clinical	Pathway	TPC-S	Phenotype cluster + pathway
Age ≥ 65	1.18 (0.72–1.92); p = 0.513	1.11 (0.68–1.82); p = 0.665	1.09 (0.66–1.81); p = 0.726	1.11 (0.67–1.87); p = 0.680
AML (vs. MDS)	1.46 (0.88–2.41); p = 0.146	1.44 (0.87–2.39); p = 0.155	1.49 (0.90–2.46); p = 0.120	1.48 (0.88–2.51); p = 0.140
Complex karyotype	2.41 (1.17–4.97); p = 0.017	2.22 (1.09–4.51); p = 0.029	2.25 (1.06–4.74); p = 0.034	2.18 (1.06–4.48); p = 0.034
TP53 VAF (per 10%)	1.15 (1.05–1.26); p = 0.003	1.16 (1.06–1.28); p = 0.002	1.15 (1.04–1.26); p = 0.005	1.15 (1.04–1.26); p = 0.005
Favourable pathway count	—	0.57 (0.36–0.91); p = 0.017	—	0.56 (0.35–0.90); p = 0.016
TPC-S (per +1)	—	—	1.24 (1.05–1.47); p = 0.013	—
Thrombo-inflammatory cluster	—	—	—	1.34 (0.73–2.46); p = 0.337
Consumption-like cluster	—	—	—	1.83 (0.92–3.65); p = 0.084
n/events	100/68	100/68	100/68	100/68
C-index: apparent (corrected, 95% CI)	0.734 (0.717, 0.682–0.740)	0.736 (0.712, 0.680–0.743)	0.739 (0.718, 0.706–0.747)	0.739 (0.704, 0.676–0.745)

All four models were fitted on the same complete-case subset of patients with full coagulation profiling permitting cluster assignment (n = 100, events = 68) to enable direct comparison of nested specifications. Model A is the clinical baseline; Models B–D each add a candidate biomarker or phenotype dimension. Hazard ratios are shown with 95% confidence intervals and Wald p-values. The C-index row reports the apparent value followed by the optimism-corrected value and its bootstrap 95% confidence interval (1000 resamples). In Model D, neither coagulation-cluster indicator reached statistical significance after adjustment for cytogenetic, molecular and pathway covariates: the Consumption-like cluster showed a non-significant ∼1.8-fold adjusted hazard (HR, 1.83, 95% CI, 0.92–3.65, p = 0.084) and the Thrombo-inflammatory cluster was null (HR, 1.34, 95% CI, 0.73–2.46, p = 0.337). Complex karyotype, TP53 VAF, and favourable-pathway count remained the consistent independent prognostic variables across models. Adding coagulation features did not improve optimism-corrected discrimination (corrected C-index: Model A 0.717 vs. Model D 0.704). Favourable pathway = ≥1 mutation in spliceosome, chromatin or transcription-factor genes. TPC-S, Thrombo-Phenotype Coagulation Score (range 0–5).

In the full model (Model D; Figure 4C), favourable pathway count stayed protective (HR 0.56, 0.35–0.90, p = 0.016), TP53 VAF stayed adverse (HR 1.15 per 10%, 1.04–1.26, p = 0.005) and complex karyotype stayed significant (HR 2.18, 1.06–4.48, p = 0.034). The Consumption-like cluster carried a non-significant, roughly 1.8-fold adverse association (HR 1.83, 95% CI 0.92–3.65, p = 0.084), and the Thrombo-inflammatory cluster was also non-significant (HR 1.34, 0.73–2.46, p = 0.337); with both confidence intervals crossing 1, neither cluster was an independent predictor in this cohort.

Continuous dose–response (Figure 4D) showed a graded relationship between D-dimer and hazard: OS hazard rose monotonically across D-dimer quintiles relative to Q1, reaching about 4-fold in the top quintile (HR ≈ 4.1 vs. Q1). A log-linear Cox model gave a hazard ratio per doubling of D-dimer of 1.18 (p = 0.003). A cubic specification suggested mild non-linearity (quadratic term p = 0.032), driven mainly by the step between the first and second quintiles (HR 1.00–2.82), but the ordering across quintiles remained monotonic. The TPC-S effect was directionally consistent across most subgroups, though not statistically significant in all (Supplementary Figure S2): AML (HR 1.33, p = 0.014), complex-karyotype (HR 1.24, p = 0.013) and multi-hit (HR 1.24, p = 0.011) patients; the association did not reach significance in MDS (HR 1.22, p = 0.093) or in those carrying favourable co-mutations (HR 1.28, p = 0.092), where the point estimates were comparable but the confidence intervals wider.

### Subgroup analysis by disease type (AML vs. MDS)

The AML and MDS subsets were also compared directly (Supplementary Table S1). As expected from the diagnostic definitions, patients with AML were younger (median 61 vs. 67 years, p = 0.002) and had a far higher marrow blast percentage (48% vs. 5.6%, p < 0.001). The two subsets were otherwise closely matched for the features that carry prognostic weight in TP53-mutated disease: complex karyotype (73.1% vs. 72.7%), multi-hit (V2) status (75.0% vs. 80.0%), TP53 VAF (medians 52.7% and 48.0%) and the presence of at least one favourable co-mutation pathway (36.5% vs. 34.5%) did not differ significantly (all p > 0.4). Among the coagulation variables only D-dimer separated the two diagnoses, being about 2.5-fold higher in AML (median 1.90 vs. 0.70 μg/mL, p = 0.014); fibrinogen, INR, PT, APTT and platelet count were essentially superimposable (all p ≥ 0.43). The distribution of the three coagulation phenotypes was likewise similar (AML: Silent 46.9%, Thrombo-inflammatory 36.7%, Consumption-like 16.3%; MDS: 54.9%, 25.5% and 19.6%, respectively; p = 0.477, calculated on the 49 AML and 51 MDS patients with an assignable cluster). Median OS was 9 months in AML and 13 months in MDS, with a comparable proportion of deaths (71.2% vs. 63.6%, p = 0.407) and no significant difference in survival between the two diagnoses (p = 0.197). Apart from the expected differences in age and blast percentage, the two diagnostic groups were therefore well balanced for the covariates entered into the multivariable models, and the higher D-dimer of the AML subset was the only coagulation feature that distinguished them.

## Discussion

In this cohort of 107 TP53-mutated AML and MDS patients, four major patterns were observed. Standard ISTH criteria detected only a fraction of coagulation activation: overt DIC was present in just 15% of cases, whereas subclinical activation, reflected by increased D-dimer, normal or elevated fibrinogen, and modestly prolonged INR, was the typical presentation at diagnosis. This activation scaled with the genomic-instability gradient, rising with complex karyotype, multi-hit TP53 status and TP53 VAF. Unsupervised clustering of routine coagulation values then separated patients into three phenotypes with a graded, statistically non-significant gradient in molecular composition and a stepwise difference in survival: Silent, Thrombo-inflammatory and Consumption-like. The prognostic weight of these phenotypes, however, was largely carried by genomic and co-mutation context. In the integrated model the Consumption-like phenotype was no longer significant once we adjusted for complex karyotype, TP53 VAF and favourable co-mutation pathway count (HR 1.83, 95% CI 0.92–3.65, p = 0.084), and the intermediate Thrombo-inflammatory phenotype was also non-significant (HR 1.34, p = 0.337). Favourable co-mutations (spliceosome, chromatin remodellers and transcription factors) were independently protective (HR 0.56, p = 0.016), while complex karyotype and TP53 VAF remained independent adverse predictors. In short, although coagulation phenotype follows the genomic-instability gradient and identifies the worst-prognosis group, it added little prognostic information beyond established cytogenetic and molecular features. This fits a picture in which coagulation activation is a correlate of TP53-driven leukaemic biology rather than a prognostic axis in its own right (Guo et al., 2020).

Our findings should also be read against the growing, AML-specific evidence that ISTH-based DIC assessment behaves differently depending on the clinical endpoint. For bleeding, the original ISTH score has limited predictive performance in non-APL AML, and a modified ISTH-based approach has been proposed to improve prediction of DIC-related bleeding (Orhan et al., 2025). For thrombosis, DIC at AML diagnosis is a strong predictor of both venous and arterial events (Libourel et al., 2016) and separate simplified scores for bleeding and for thrombosis have been developed for newly diagnosed acute leukaemia (Owattanapanich et al., 2023). Because the present study did not capture bleeding or thrombotic events, the coagulation phenotypes and the TPC-S described here should be understood as survival- and biology-associated laboratory patterns, not as clinically validated tools for predicting bleeding or thrombosis; prospective cohorts that record these events will be required before any endpoint-specific clinical use can be considered.

A central contribution of this work is the biological interpretation of the three coagulation phenotypes. The Silent phenotype probably signifies early-stage or low-burden disease, perhaps analogous to clonal haematopoiesis that precedes overt leukemia, or representing cases where genomic instability has not yet initiated inflammatory or coagulation pathways (Young et al., 2016). Conversely, the Thrombo-inflammatory phenotype, marked by a pronounced increase in fibrinogen but only moderate D-dimer elevation, suggests an acute-phase inflammatory reaction superimposed on leukemia, likely mediated by cytokine release from aneuploid blasts or by subclinical infection at diagnosis (Strasser et al., 2025).The Consumption-like phenotype, defined by globally prolonged coagulation times, low platelets and the highest TP53 VAF (69.5%) (Punwani and Chhabra, 2025), is consistent with a sub-clinical consumptive coagulopathy at an advanced stage of genomic instability. The proportion of MDS in this cluster (56%) was, however, no higher than in the Silent cluster (55%) or in the cohort as a whole (51%; p = 0.477), so the phenotype cannot be attributed to disease category. Although it carries an approximately two-fold crude hazard, its adjusted association did not reach significance (HR 1.83, p = 0.084), and on current evidence it is best read as a marker of cytogenetic and molecular severity rather than a source of independent prognostic information. These interpretations remain hypothetical and will need mechanistic validation in independent cohorts with paired cytokine and microvesicle assays. Because no cytokine, tissue factor, microvesicle, or cGAS–STING assays were performed here, these mechanistic links are hypotheses generated from routine laboratory patterns rather than demonstrated findings.

The signal associated with chromatin pathway alterations (HR 0.46, p = 0.030) was among the most striking findings, although its interpretation is limited by the small number of affected patients (n = 21) and should be considered exploratory pending external validation. The observation is nonetheless supported by the consistent direction of effect across spliceosome and transcription-factor co-mutations, as well as by the significant cumulative benefit associated with increasing numbers of favourable pathways (HR 0.52 per pathway, p = 0.003). One possibility is that these co-mutations identify a biologically distinct subset of TP53-mutated disease. Previous studies have suggested that TP53-mutated MDS with SF3B1 co-mutation may exhibit a more indolent phenotype (Malcovati et al., 2015), whereas TP53-mutated AML retaining transcription-factor lesions may preserve partial differentiation programmes (Papaemmanuil et al., 2016). The absence of a significant interaction between favourable pathway count and TPC-S argues against a synergistic relationship and instead suggests that molecular context and coagulation status contribute independently to outcome, reflecting separate aspects of disease biology.

The frequent occurrence of subclinical coagulation activation in TP53-mutated AML/MDS suggests that routine measurement of D-dimer and fibrinogen at diagnosis may have value beyond the detection of overt DIC. These inexpensive and widely available assays may provide additional insight into disease biology, particularly in patients exhibiting the Consumption-like phenotype. Notably, this subgroup experienced a median survival of only 8 months, highlighting its adverse clinical course. It must be emphasised, however, that this value is biological rather than immediately clinical: the Consumption-like phenotype lost independent prognostic significance after multivariable adjustment (HR 1.83, 95% CI 0.92–3.65, p = 0.084), and adding coagulation information to the clinical baseline did not improve optimism-corrected discrimination (C-index 0.717 vs. 0.704). Any clinical application of coagulation profiling in this setting therefore remains speculative and contingent on prospective validation. Whether such patients derive particular benefit from therapies targeting genomic-instability-associated inflammatory signalling remains uncertain, but they may constitute an attractive population for studies evaluating cGAS–STING-directed strategies and other approaches aimed at innate immune activation (Diepstraten et al., 2024). The TPC-S (range 0–5) retained independent prognostic relevance after accounting for established disease features, including karyotype and TP53 VAF (HR 1.24 per point, p = 0.013), and showed a consistent directional effect across major patient subsets. These findings suggest that coagulation-derived metrics may capture aspects of disease biology not fully reflected by current molecular and cytogenetic classifiers; this did not, however, translate into better discrimination, as adding the TPC-S to the clinical baseline left the optimism-corrected C-index essentially unchanged (Model C 0.718 vs. Model A 0.717). Accordingly, TPC-S is best viewed as a complementary tool rather than a substitute for existing risk-stratification frameworks, with the advantage of being readily implementable in settings where routine coagulation testing is already performed.

### Limitations

This study has several limitations. Its retrospective, single-center design raises the usual concerns regarding generalisability and potential unmeasured confounding, including cause-specific mortality, treatment intensity, baseline infection burden, and supportive-care practices. Although relatively large for a TP53-mutated AML/MDS cohort, the sample size (n = 107) remains limited, particularly for the Consumption-like cluster (n = 18), which did not reach statistical significance in Model D (HR 1.83, 95% CI 0.92–3.65, p = 0.084), leaving its independent prognostic contribution uncertain. Multi-hit TP53 status was inferred indirectly, as centralized 17p assessment was not uniformly available, and the surrogate used (complex karyotype or VAF >50%) is biologically plausible but imperfect (Shah et al., 2025). Adding coagulation features to the clinical baseline did not improve optimism-corrected discrimination (C-index 0.717 vs. 0.704), underscoring that the primary contribution of this work is the biological insight into TP53-associated coagulation phenotypes rather than enhanced survival prediction. Thrombotic and hemorrhagic events were not analyzed due to inconsistent documentation, representing an important focus for future prospective studies. Finally, although the clustering was internally coherent, it should be considered exploratory until validated in an independent cohort with comparable pre-treatment coagulation profiling. Several further caveats deserve emphasis. First, although AML and high-risk MDS share TP53-driven biology, they differ in treatment and in baseline haemostatic profile, so pooling them may introduce disease-type confounding despite covariate adjustment and the pre-specified subgroup and interaction testing, which were not powered to exclude modest between-disease differences. Second, the unsupervised phenotypes warrant particular caution: the modest average silhouette width (0.22) indicates limited between-cluster separation, no external validation cohort was available, and cluster stability was assessed only internally by bootstrap resampling rather than in independent data; the three phenotypes are therefore best viewed as a hypothesis-generating description of coagulation heterogeneity rather than as biologically discrete entities, and their biological significance should not be overstated. Finally, the retrospective nature of this analysis precludes causal inference.

## Conclusion

In conclusion, activation of coagulation in TP53-mutated AML/MDS appears to be associated with the extent of underlying genomic-instability-related features. The three coagulation phenotypes identified in this study, namely, Silent, Thrombo inflammatory, and Consumption like, may provide a more refined characterization of disease heterogeneity than conventional DIC-based classifications. Among these groups, patients with the Consumption like phenotype showed the poorest survival outcomes. However, this association was no longer statistically significant after adjustment for genomic variables, and the phenotype did not remain an independent prognostic factor in our cohort. Nevertheless, it may still offer important insights into the inflammatory and procoagulant processes associated with TP53 deficiency. Future prospective multicenter studies, incorporating parallel analyses of cytokines and microvesicles together with detailed recording of thrombotic and bleeding events, are needed to confirm these findings before they can be considered for clinical application.

## Data Availability

The datasets generated and/or analyzed during the current study are not publicly available due to patient privacy and institutional ethical restrictions. De-identified data may be made available upon reasonable request to the corresponding author, subject to approval and compliance with institutional ethical and data protection requirements.

## References

[B1] Bernard E. Nannya Y. Hasserjian R. P. Devlin S. M. Tuechler H. Medina-Martinez J. S. (2020). Implications of TP53 allelic state for genome stability, clinical presentation and outcomes in myelodysplastic syndromes. Nat. Med. 26, 1549–1556. 10.1038/s41591-020-1008-z 32747829 PMC8381722

[B2] Bernard E. Hasserjian R. P. Greenberg P. L. Arango Ossa J. E. Creignou M. Tuechler H. (2024). Molecular taxonomy of myelodysplastic syndromes and its clinical implications. Blood 144, 1617–1632. 10.1182/blood.2023023727 38958467 PMC11487646

[B3] Daver N. G. Maiti A. Kadia T. M. Vyas P. Majeti R. Wei A. H. (2022). TP53-Mutated myelodysplastic syndrome and acute Myeloid leukemia: biology, current therapy, and future directions. Cancer Discov. 12 (11), 2516–2529. 10.1158/2159-8290.CD-22-0332 36218325 PMC9627130

[B4] Diepstraten S. T. Yuan Y. La Marca J. E. Young S. Chang C. Whelan L. (2024). Putting the STING back into BH3-mimetic drugs for TP53-mutant blood cancers. Cancer Cell 42, 850–868. 10.1016/j.ccell.2024.04.004 38670091

[B5] Ding Y. Qi X. Li Y. Sun Y. Wan J. Luo C. (2023). Albumin-to-fibrinogen ratio is an independent prognostic parameter in *de novo* non-M3 acute myeloid leukemia. Clin. Exp. Med. 23, 4597–4608. 10.1007/s10238-023-01241-8 37914966

[B6] Falanga A. Marchetti M. Vignoli A. (2013). Coagulation and cancer: biological and clinical aspects. J. Thrombosis Haemostasis Jth 11, 223–233. 10.1111/jth.12075 23279708

[B7] Geddings J. E. Mackman N. (2013). Tumor-derived tissue factor-positive microparticles and venous thrombosis in cancer patients. Blood 122, 1873–1880. 10.1182/blood-2013-04-460139 23798713 PMC3772497

[B8] Grob T. Al Hinai A. S. A. Sanders M. A. Kavelaars F. G. Rijken M. Gradowska P. L. (2022). Molecular characterization of mutant TP53 acute myeloid leukemia and high-risk myelodysplastic syndrome. Blood 139, 2347–2354. 10.1182/blood.2021014472 35108372 PMC11022827

[B9] Guo Z. Chen X. Tan Y. Xu Z. Xu L. (2020). Coagulopathy in cytogenetically and molecularly distinct acute leukemias at diagnosis: comprehensive study. Blood Cells, Mol. and Dis. 81, 102393. 10.1016/j.bcmd.2019.102393 31918382

[B10] Horowitz N. A. Brenner B. (2020). Thrombosis in hematological malignancies: mechanisms and implications. Thromb. Res. 191 (Suppl. 1), S58–S62. 10.1016/S0049-3848(20)30398-4 32736780

[B11] Kumar A. Kim C. Hoffman T. A. Naqvi A. Dericco J. Jung S. (2011). p53 impairs endothelial function by transcriptionally repressing kruppel-like factor 2. Arteriosclerosis, Thrombosis, Vasc. Biol. 31, 133–141. 10.1161/ATVBAHA.110.215061 20947822 PMC3064482

[B12] Libourel E. J. Klerk C. P. W. van Norden Y. de Maat M. P. M. Kruip M. J. Sonneveld P. (2016). Disseminated intravascular coagulation at diagnosis is a strong predictor for thrombosis in acute myeloid leukemia. Blood 128, 1854–1861. 10.1182/blood-2016-02-701094 27354723

[B13] Malcovati L. Karimi M. Papaemmanuil E. Ambaglio I. Jädersten M. Jansson M. (2015). SF3B1 mutation identifies a distinct subset of myelodysplastic syndrome with ring sideroblasts. Blood 126, 233–241. 10.1182/blood-2015-03-633537 25957392 PMC4528082

[B14] Orhan B. Özkalemkaş F. Bayır T. Yalçın C. Güner B. Elgün E. (2025). Predicting bleeding in AML-Associated DIC: limitations of the ISTH Score and a modified approach. Diagn. (Basel) 15 (23), 3053. 10.3390/diagnostics15233053 41374437 PMC12692086

[B15] Owattanapanich W. Rungjirajittranon T. Jantataeme A. Kungwankiattichai S. Ruchutrakool T. (2023). Simplified predictive scores for thrombosis and bleeding complications in newly diagnosed acute leukemia patients. Thromb. J. 21 (1), 65. 10.1186/s12959-023-00506-2 37291589 PMC10251548

[B16] Papaemmanuil E. Gerstung M. Bullinger L. Gaidzik V. I. Paschka P. Roberts N. D. (2016). Genomic classification and prognosis in Acute Myeloid leukemia. N. Engl. J. Med. 374, 2209–2221. 10.1056/NEJMoa1516192 27276561 PMC4979995

[B17] Punwani N. Chhabra S. (2025). Curing the incurable: TP53 mutated Myeloid neoplasms. Clin. Lymphoma, Myeloma and Leukemia 25, e976–e985. 10.1016/j.clml.2025.07.013 40849278

[B18] Rickles F. R. Falanga A. (2009). Activation of clotting factors in cancer. Cancer Treat. Res. 148, 31–41. 10.1007/978-0-387-79962-9_3 19377917

[B19] Rücker F. G. Schlenk R. F. Bullinger L. Kayser S. Teleanu V. Kett H. (2012). TP53 alterations in acute myeloid leukemia with complex karyotype correlate with specific copy number alterations, monosomal karyotype, and dismal outcome. Blood 119, 2114–2121. 10.1182/blood-2011-08-375758 22186996

[B20] Shah M. V. Hung K. Baranwal A. Wechalekar G. Al-Kali A. Toop C. R. (2025). Validation of the. Blood Cancer J. 15, 88. 5th edition of the World Health Organization and International Consensus Classification guidelines for TP53-mutated myeloid neoplasm in an independent international cohort. 10.1038/s41408-025-01290-0 40335478 PMC12059121

[B21] Strasser B. Mustafa S. Seier J. Tomasits J. Haushofer A. (2025). “Hematopathological patterns in Acute Myeloid leukemia with complications of overt disseminated intravascular coagulation,” Diagnostics 15. 383. 10.3390/diagnostics15030383 39941313 PMC11817403

[B22] Tashakori M. Kadia T. Loghavi S. Daver N. Kanagal-Shamanna R. Pierce S. (2022). TP53 copy number and protein expression inform mutation status across risk categories in acute myeloid leukemia. Blood 140, 58–72. 10.1182/blood.2021013983 35390143 PMC9346958

[B23] Taylor F. B. J. Toh C. H. Hoots W. K. Wada H. Levi M. Scientific SODI (2001). Towards definition, clinical and laboratory criteria, and a scoring system for disseminated intravascular coagulation. Thromb. Haemost. 86, 1327–1330. 10.1055/s-0037-1616068 11816725

[B24] Tazi Y. Arango-Ossa J. E. Zhou Y. Bernard E. Thomas I. Gilkes A. (2022). Unified classification and risk-stratification in Acute Myeloid Leukemia. Nat. Commun. 13, 4622. 10.1038/s41467-022-32103-8 35941135 PMC9360033

[B25] Welch J. S. Petti A. A. Miller C. A. Fronick C. C. O'Laughlin M. Fulton R. S. (2016). TP53 and decitabine in Acute Myeloid leukemia and myelodysplastic syndromes. N. Engl. J. Med. 375, 2023–2036. 10.1056/NEJMoa1605949 27959731 PMC5217532

[B26] Yokoyama M. Shimizu I. Nagasawa A. Yoshida Y. Katsuumi G. Wakasugi T. (2019). p53 plays a crucial role in endothelial dysfunction associated with hyperglycemia and ischemia. J. Mol. Cell Cardiol. 129, 105–117. 10.1016/j.yjmcc.2019.02.010 30790589

[B27] Young A. L. Challen G. A. Birmann B. M. Druley T. E. (2016). Clonal haematopoiesis harbouring AML-associated mutations is ubiquitous in healthy adults. Nat. Commun. 7, 12484. 10.1038/ncomms12484 27546487 PMC4996934

