# Supplementary material for: Coagulation activation is associated with genomic-instability-related features in TP53-mutated AML and MDS: routine laboratory patterns beyond classical disseminated intravascular coagulation
Source: Front Cell Dev Biol. 2026 Aug 21;14:1918027. doi: 10.3389/fcell.2026.1918027 (PMC13541715; doi:10.3389/fcell.2026.1918027)
Supplement: Supplementary file 1 [file Image1.pdf]

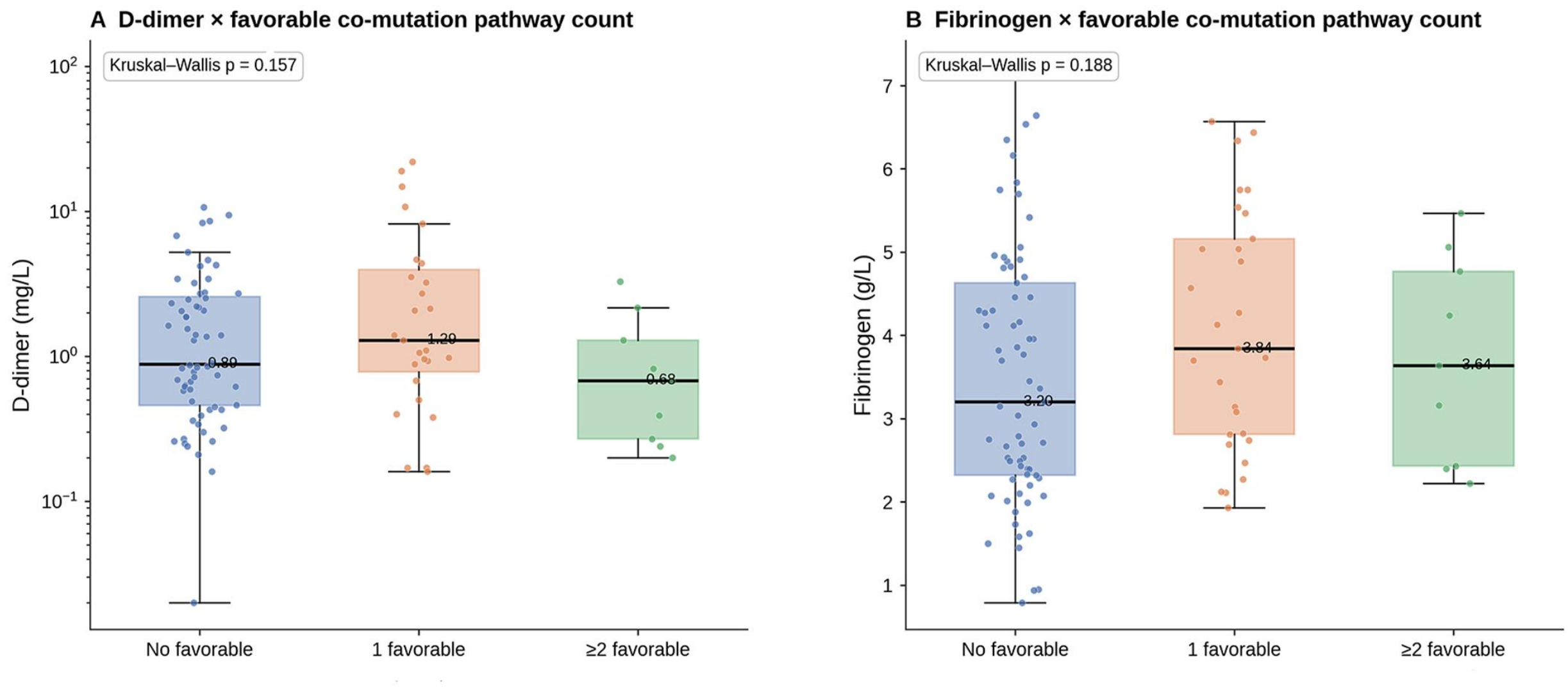

Supplementary Figure S1

Coagulation markers by favourable co-mutation pathway count. Distributions of (A) D-dimer and (B) fibrinogen according to favourable-pathway count. Boxes show medians and interquartile ranges. P values were obtained using Kruskal–Wallis tests. D-dimer and fibrinogen analyses included 100 and 107 patients, respectively.
