# Supplementary material for: Coagulation activation is associated with genomic-instability-related features in TP53-mutated AML and MDS: routine laboratory patterns beyond classical disseminated intravascular coagulation
Source: Front Cell Dev Biol. 2026 Aug 21;14:1918027. doi: 10.3389/fcell.2026.1918027 (PMC13541715; doi:10.3389/fcell.2026.1918027)
Supplement: Supplementary file 2 [file Image2.pdf]

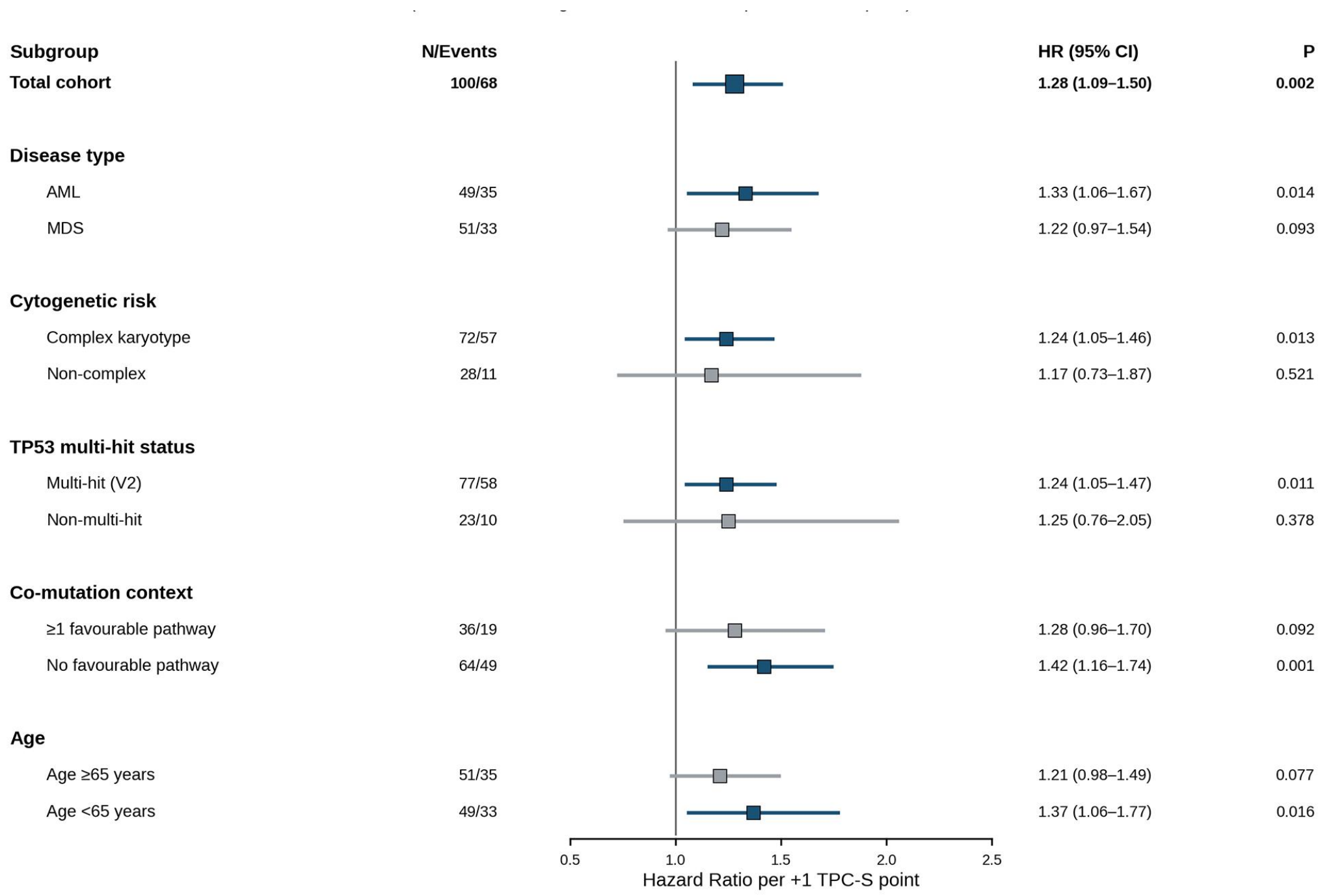

Supplementary Figure S2

Subgroup associations between TPC-S and overall survival. Univariable Cox associations between TPC-S and OS in the complete-case cohort and selected subgroups. HRs are expressed per 1-point increase; squares and lines show HRs and 95% CIs. Blue indicates nominal $P<0.05$ and grey $P \geq 0.05$. No interaction P values are presented; differences in subgroup significance should not be interpreted as effect modification.
